# Anti-*Pseudomonasaeruginosa* activity of a C_16_-terpene dilactone isolated from the endophytic fungus *Neofusicoccumluteum* of *Kigeliaafricana* (Lam.)

**DOI:** 10.1038/s41598-021-04747-x

**Published:** 2022-01-17

**Authors:** Olusola Bodede, Mamokoena Kuali, Gerhard Prinsloo, Roshila Moodley, Roshini Govinden

**Affiliations:** 1 School of Chemistry and Physics, University of KwaZulu-Natal, Westville Campus, Private Bag X54001, Durban, 4000 South Africa; 2 School of Life Sciences, University of KwaZulu-Natal, Westville Campus, Private Bag X54001, Durban, 4000 South Africa; 3 Department of Agriculture and Animal Health, University of South Africa, Florida Campus, Florida, 1710 South Africa

**Keywords:** Chemistry, Medicinal chemistry, Organic chemistry, Microbiology, Antimicrobials, Fungi

## Abstract

Fungal endophytes have the capacity to biosynthesize secondary metabolites that are produced by their host plants. In this study, a dilactone terpenoid of C_16_ architecture was isolated from the fungal endophytes of *Kigelia* *africana*, in our attempt to identify anti-*Pseudomonas* *aeruginosa* metabolites. Thirty-eight fungal isolates were cultured for biomolecule production over a period of thirty days. Extracts from three (ZF 34, ZF 52 and ZF 91) of the fungi showed good anti-*P.* *aeruginosa* activity, with ZF 52 presenting the best MIC of 19.53 µg/mL and was accordingly subjected to chromatographic separation. Based on nuclear magnetic resonance (NMR) spectroscopy, high resolution mass spectrometry and single crystal X-ray diffraction (XRD) analyses, the isolated compound was identified as a C_16_-terpene dilactone, with a structure consistent with that of the known diterpene, CJ-14445. The isolated dilactone showed anti-*P.* *aeruginosa* activity with MIC of 0.61 µg/mL, signifying the antibacterial potential of the biomolecule. The bioactive fungal isolate (ZF 52) was identified as *Neofusicoccum* *luteum* based on genomic DNA sequencing. This is the first report of the endophyte *N.* *luteum* from *K.* *africana* and the first reported occurrence of CJ-14445 in the fungus.

## Introduction

It has been established that fungal endophytes can biosynthesize bioactive secondary metabolites that are produced by their host plants^1^. The differences observed in the production of bioactive metabolites by plants investigated across different geographical locations suggest that plant microbiomes may be responsible for producing certain phytocompounds and not the plants^1,2^. Thus, the wide diversity of fungal endophytes has the potential to provide an *in-vitro* culture-based alternative and sustainable source of bioactive compounds required to meet the rising demands on natural products for antibiotic drug discovery^1,3^.

*Neofusicoccum* *luteum* is a typical species of the fungi family Botryosphaeriaceae, commonly known as a pathogen associated with leaf necrosis and fruit rot of olives in Australia^4^, avocado branch canker in California^5^, dieback on hybrid Rhododendrons^6^ and grapevines^7^. More recently, there have been reports on isolation of *Neofusicoccum* sp. endophytes from mangrove^8^, eucalyptus^9^ and fruit trees^10^. Although these authors did not investigate secondary metabolites produced by these isolates, there are recent reports on compounds associated with *Neofusicoccum*, most of which are phytotoxic with few having antibacterial, antiproliferative and anti-atherosclerotic activities^11,12^. The compounds belong to classes such as cyclohexenones, pyrones, melleins, naphthalenones, naphthoquinones, phenols, fatty acids and sesquiterpenes, with botryosterpene being the only reported C-_16_ tetracyclic member^12^. Several skeletally similar compounds have been isolated from *Botryosphaeria* sp. P483^13^ and other fungi^14,15^. These include the C-_16_ dilactone, CJ-14445 and it’s analogs, often classified as tetranorditerpenoid dilactones or oidiolactones (being common *Oidiodendron* metabolites), some of which have found application as antimicrobial^13,16^ antiplasmodial and herbicidal^15^.

Extracts of *K.* *africana*’s fungal endophytes have displayed significant antimicrobial activity^17^ against the human pathogenic bacterium, *Pseudomonas* *aeruginosa*. However, there is insufficient information on the secondary metabolites available in the plant’s mycobiome.

*P.* *aeruginosa* is an aerobic, rod-shaped Gram-negative bacterium with multifaceted pathogenicity, causing about 10% of hospital infections, including chronic lung infections that lead to the death of patients with cystic fibrosis. It is highly adaptive, as evidenced by its ability to survive the stressful environment of a lung with cystic fibrosis^18^. It tolerates a temperature of 4–42 °C and is often referred to as an opportunistic pathogen due to its ability to cause diseases in immuno-compromised patients^19^. Herein, we aim to identify the endophytic fungal strain from *K.* *africana* with significant anti-*P.* *aeruginosa* activity using molecular methods and to isolate and identify the bioactive compounds from the fungus.

## Results

### Fungal culture for biomolecule production

**Figure 1:**
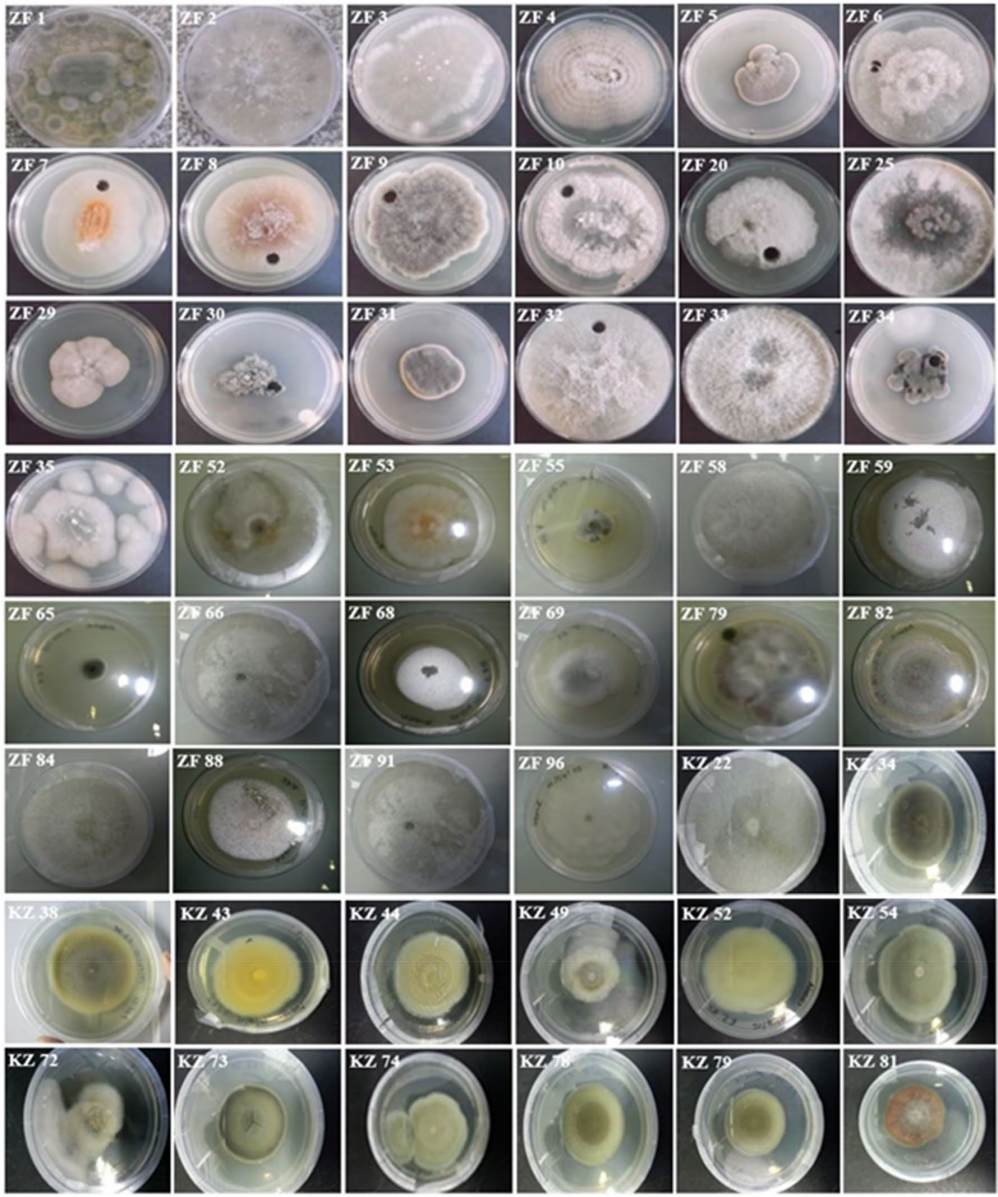
Pure fungal cultures identified from *Kigella* *africana* leaves.

**Figure 2:**
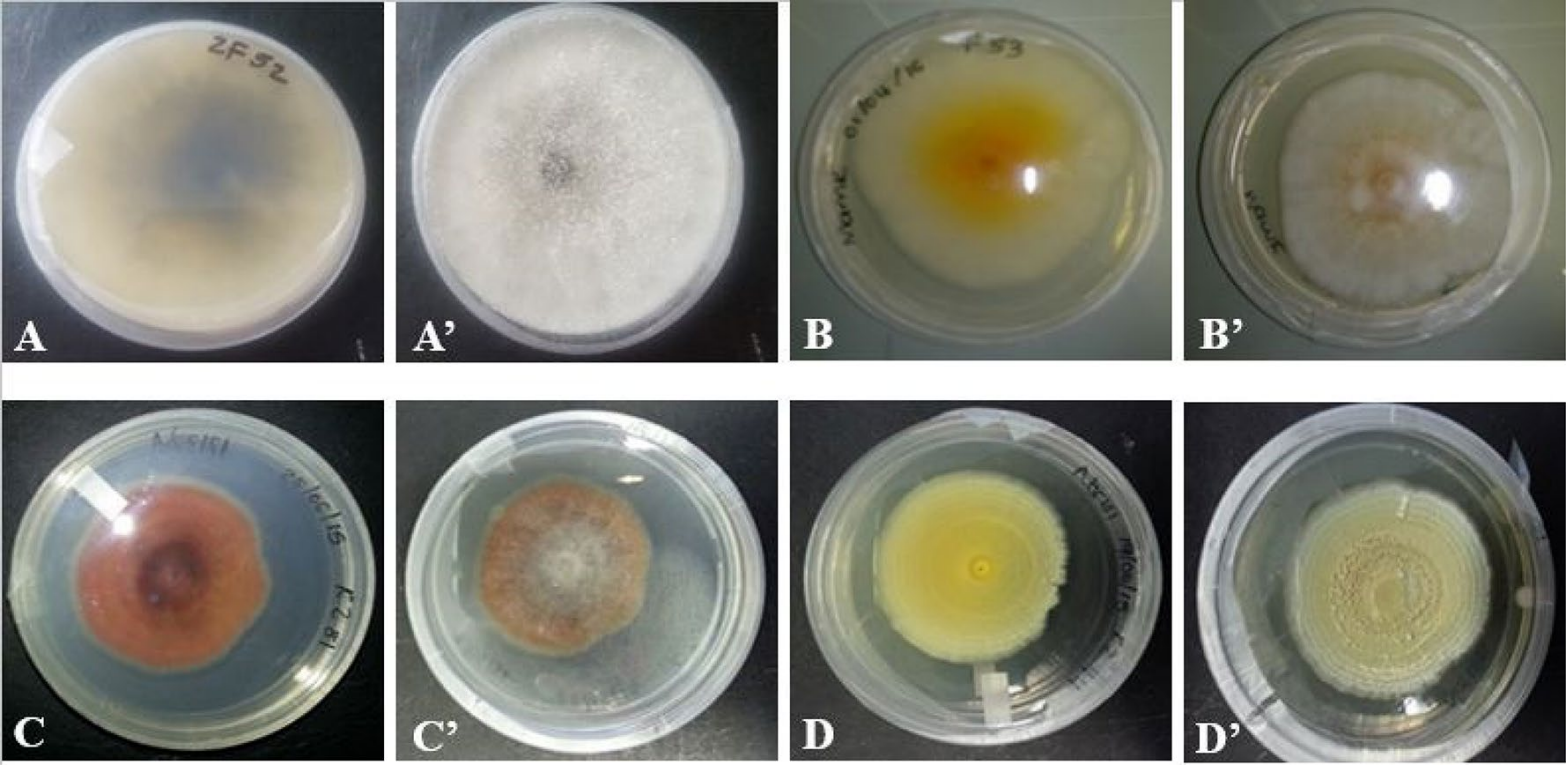
Growth of endophytic fungi upon resuscitation on potato dextrose agar media for four days. Top view of ZF 52, ZF 53, KZ 81 & KZ 44 are represented by (**A**–**D**) respectively while bottom views are represented by (**A’**–**D’**), respectively.

Forty-eight pure fungal isolates were morphologically identified from *K.* *africana* leaves (Fig. 1). Pigmentations such as white, cream, yellow, green and black as well as structural variations of colonies were used to differentiate between the different isolates. Potato dextrose agar plates were used to successfully resuscitate thirty-three endophytic fungi as the grown fungi matched the original isolates in terms of varying characteristics and pigmentation. The pigmentation of the mycelia intensified with age from lightly pigmented as the mycelia grew into fresh media (Fig. 2). This was observed for all fungi growing on PDA. The solid substrate fermentation reaction contents showed varying pigmentation, where initial growth was observed on day four of the 30-day period for fast growing fungi and as delayed at day 15 for slow growing fungi. The presence of white fluffy growths on the surface of the solid substrate was present in all the fermentation vessels. A characteristic of most of the fermenting biomass was that it matched the pigment observed on PDA plates, except for ZF 52, which was both black and yellow on PDA and only black in SSF.

### Anti-Pseudomonas aeruginosa activity

**Figure 3:**
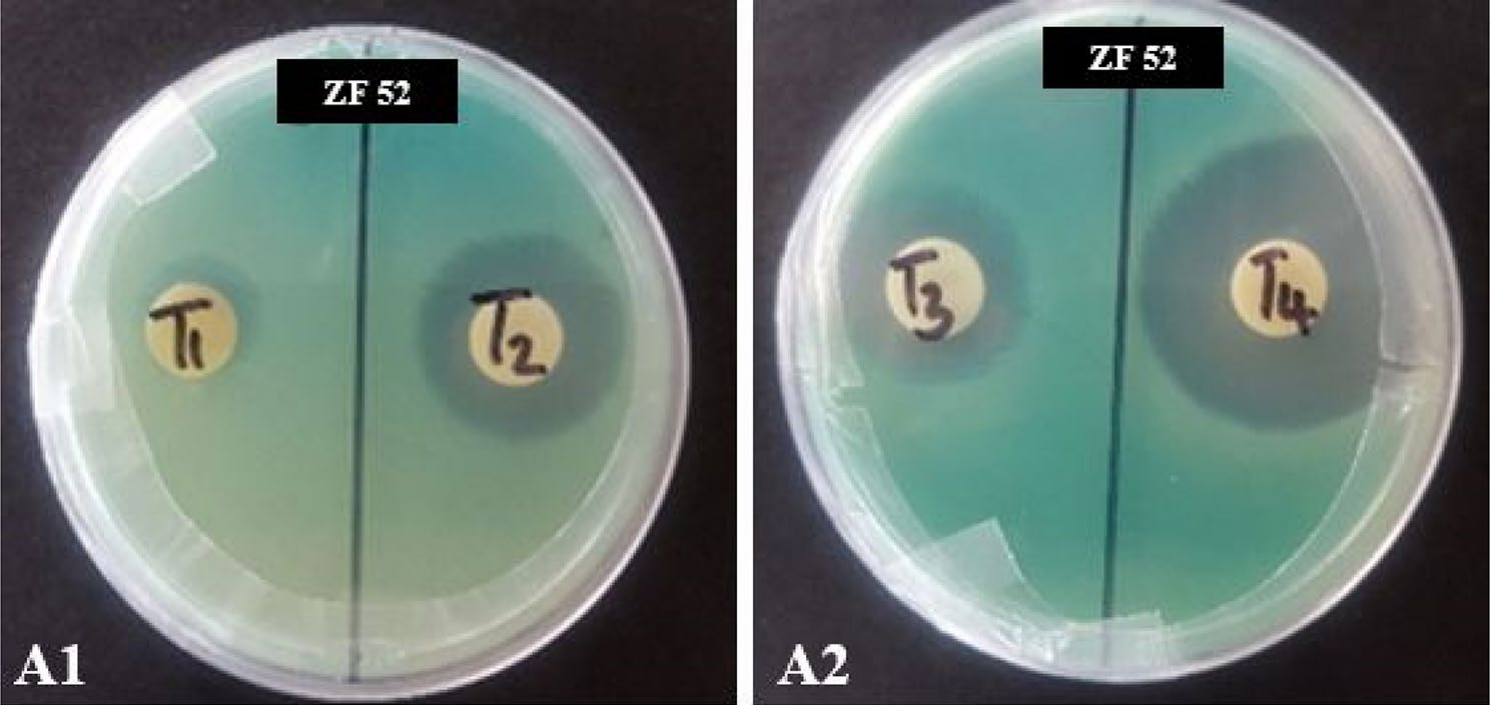
Kirby Bauer disc diffusion assay demonstrating anti-*Pseudomonas* *aeruginosa* activity of ZF 52 extracts after varying fermentation periods (T1, T2, T3, and T4 represent 1, 2, 3 and 4 weeks, respectively).

Variable results were obtained using the disc diffusion assay for the 48 extracts. Most extracts did not inhibit *P.* *aeruginosa*, whilst ZF 34, ZF 52 and ZF 91 exhibited significant activity with zones of inhibition ranging from 0–40 mm. However, due to challenges experienced in resuscitating long-term cultures for ZF 34 and ZF 91 from storage, they were eliminated from further testing leaving ZF 52 (which also gave the largest zone of inhibition in the preliminary test) as the only fungal extract to be tested further. A time course was conducted in order to observe the difference in activity from week one (T1) to week four (T4) (Fig. 3). The MICs of the ZF 52 extracts were 156.5 µg/mL, 39.06 µg/mL, 78.73 µg/mL and 19.53 µg/mL for T1, T2, T3 and T4, respectively as given in Table 1. This suggests that crude fungal extracts develop greater antimicrobial activity over time as the highest anti-*P.* *aeruginosa* activity was obtained after 4 weeks of fungal culture.

**Table 1:** Minimum inhibitory concentrations (MICs) of ZF 52 crude and semi purified extracts and compound **1** (3a,10b-dimethyl-1,2,3,3a,5a,7,10b,10c-octahydro-5,8-dioxa-acephenanthrylene-4,9-dione) against *P.* *aeruginosa*.

Extracts/compound	Fungal age	MIC (µg/mL)
ZF 52	T1	156.50
ZF 52	T2	39.06
ZF 52	T3	78.73
ZF 52	T4	19.53
Fraction C (partially purified ZF 52)	N/A	2.44
Compound 1	N/A	0.61
Gentamycin	N/A	4.00

Gentamycin served as the positive control.

T1, T2, T3, and T4 represent weeks 1, 2, 3 and 4, respectively in the fungal culture period. N/A = not applicable.

### Identification of isolated compound

**Figure 4:**
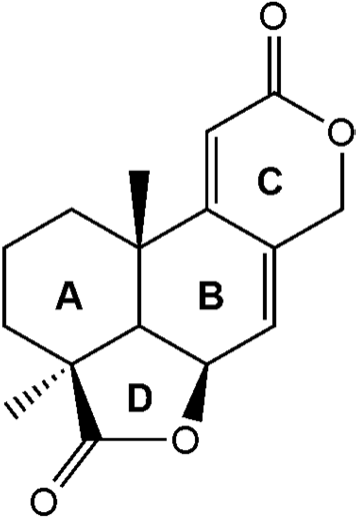
Structure of compound **1**.

A solvent system, hexane/EtOAc (ethyl acetate) was used to develop ZF 52 extract in a thin layer chromatographic procedure. Compound **1** was obtained as a major metabolite in the extract with retention factor 0.78. Compound **1** (35 mg) was obtained as a white crystalline solid from the EtOAc extract of ZF 52. The mass spectral data for Compound **1** is as follows; HR-ESI–MS: *m/z* 297.1109 [M + Na]^+^, (calcd for C_16_H_18_O_4_Na, 297.1103). IR (KBr) *ν*_max_ cm^-1^: 2926, 2879, 1771, 1702, 1612, 1466, 1384, 1200, 1034, 878. The NMR spectroscopic data of **1** is presented in Table 2 while the structure is given in Fig. 4. The absolute configuration of compound **1** was determined by single crystal X-ray diffraction (XRD) analysis. All NMR, FTIR spectra, High Resolution Mass Spectral report and XRD reports of **1** are provided in the supplementary material.

**Table 2:** NMR (400 MHz) spectral data of compound **1** recorded in CDCl_3_.

Position	δ_H_ (multiplicity; *J* in Hz)	δ_C_
1	1.72–1.58^a^	29.6
2	1.72–1.58^a^	17.3
3	2.24 (ddd; 13.71, 7.66, 5.64) 1.53 (ddd; 14.28, 7.45, 6.06)	27.7
4		42.7
5	1.92 (d; 4.66)	47.8
6	5.00 (brt; 4.52)	71.3
7	6.18 (m)	121.8
8		132.2
9		158.7
10		35.0
11	5.73 (d; 1.52)	111.7
12		163.6
13	4.96 (dt; 13.57) 4.87 (d; 13.57)	69.5
14		180.8
15	1.30 (s)	24.1
16	1.15 (s)	24.7

^a^Overlapping chemical shifts.

### Chemical profile of ZF 52 using Ultra Performance Liquid Chromatography-Electrospray Ionization-Mass Spectrometry (UPLC-ESI–MS)

**Figure 5:**
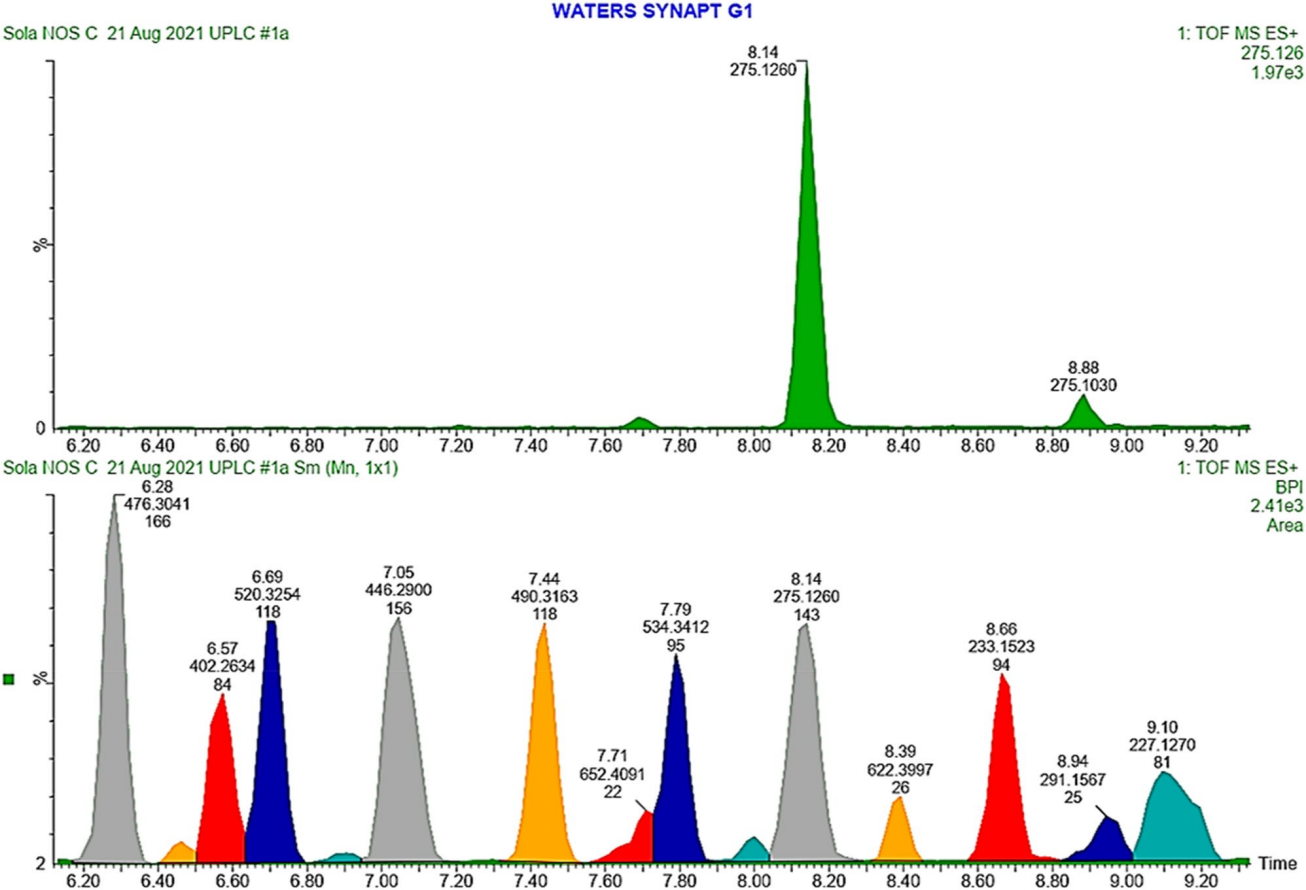
UPLC-ESI–MS fingerprint of ZF 52 EtOAc extract.

**Figure 6:**
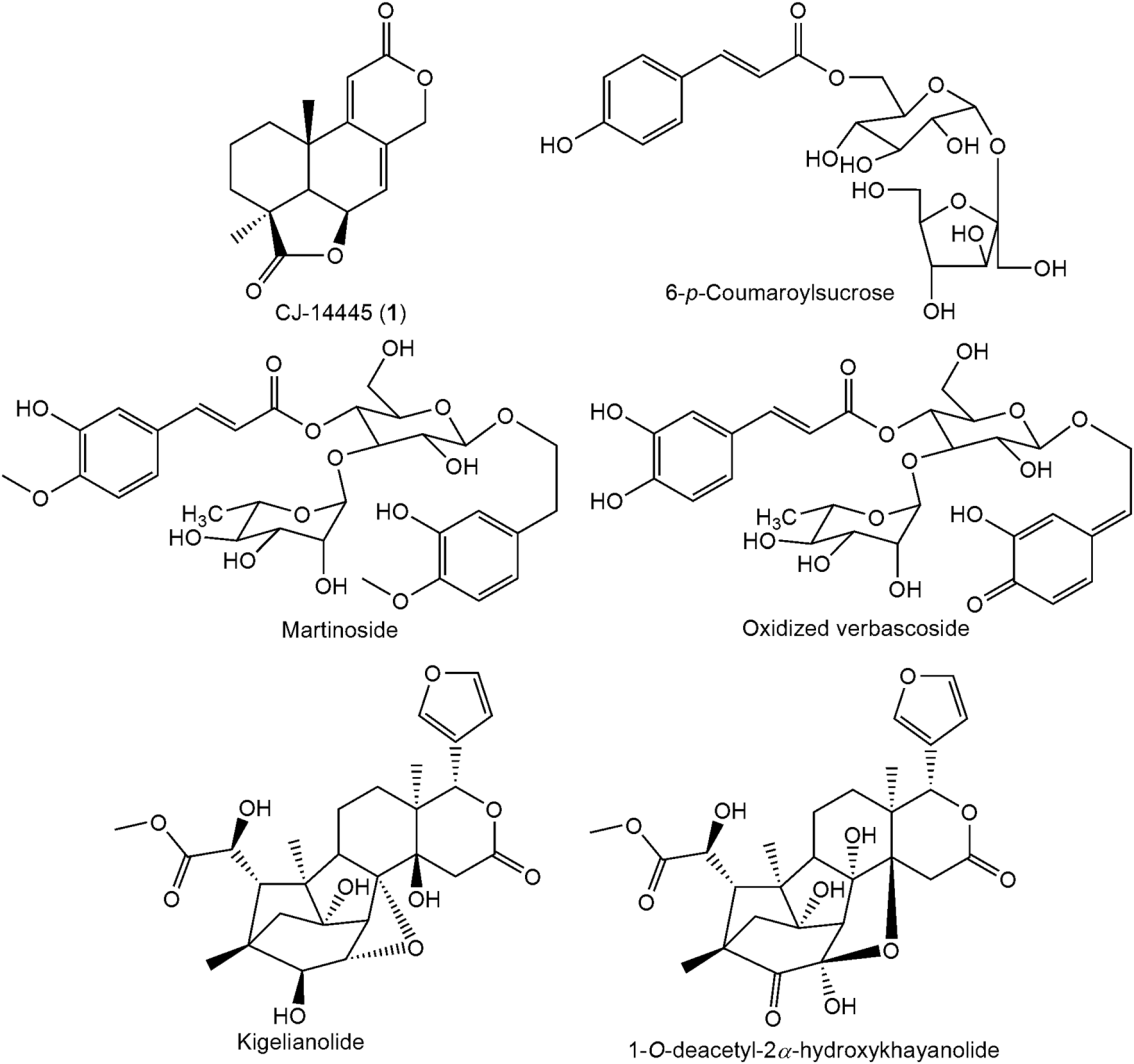
Proposed structures for compounds identified in ZF 52 ethyl acetate extract by UPLC-ESI–MS.

Twelve major peaks observed in the UPLC-ESI–MS chromatogram were quantitatively integrated and annotated (Fig. 5). The isolated compound alongside five other metabolites were identified. The reports of the compounds are provided in Table 3 while their chemical structures are given in Fig. 6.

**Table 3:** UPLC-TOF–MS report of ZF 52 EtOAc extract.

t_R_ (min)	Suggested compound	Formula	Found mass	Adduct
6.69	Kigelianolide	C_27_H_36_O_10_	520.3254	[M + 2H]^+^
7.44	6-*p*-Coumaroylsucrose	C_21_H_28_O_13_	490.3163	[M + 2H]^+^
7.71	Martinoside	C_31_H_40_O_15_	652.4091	[M]^+^
7.79	1-*O*-deacetyl-2*α*-hydroxykhayanolide E	C_27_H_32_O_11_	534.3412	[M + 2H]^+^
8.14	CJ-14445	C_16_H_18_O_4_	275.1260	[M + 1]^+^
8.39	Oxidized verbascoside	C_29_H_34_O_15_	622.3997	[M]^+^

### Genomic DNA identification

**Figure 7:**
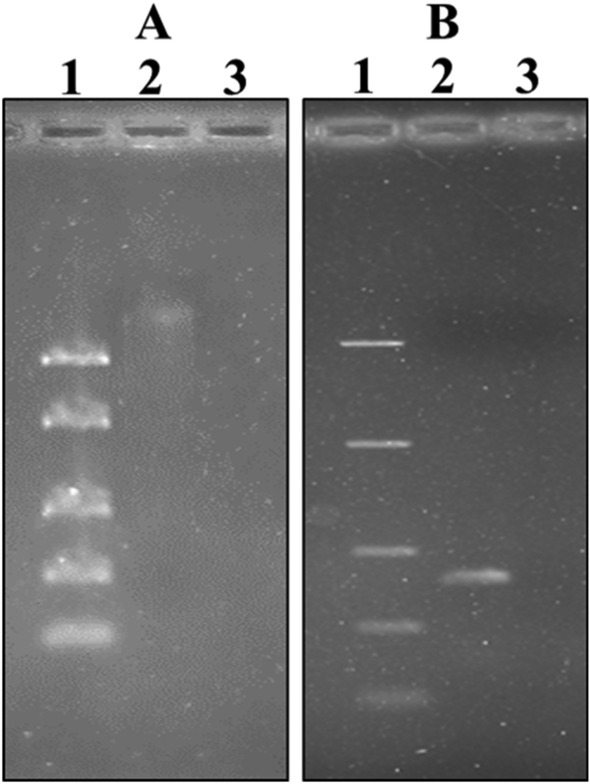
Gel electrophoresis image of ZF 52 genomic DNA. A1—Mid-range molecular weight marker, A2—ZF52 DNA, A3—negative control, B1—Mid-range molecular weight marker, B2—PCR amplification product, B3—negative control.

Limited yields of genomic DNA were obtained after several attempts of using aged mycelia of ZF 52. In the final attempt, a 24 h culture was manually disrupted using forceps. The resultant small mycelia were then subjected to bead bashing as per kit instruction manual but the vortexing time was increased from 30 to 60 s. A barely visible yield of genomic with some streaking indicating damaged DNA was able to be visualized on an agarose gel after staining with ethidium bromide (Fig. 7A). Figure 7B shows the gel image of the PCR product obtained after amplification of the ITS2 region of the 18S rRNA gene. The PCR amplification was successful as the expected amplicon of 600 bp was obtained. The 18S PCR amplicon was sequenced at Stellenbosch University, South Africa. Trimmed forward and reverse sequences were aligned and submitted to Genbank for comparison to other sequences. The result returned from Genbank showed ZF 52 has 99% identity to *N.* *luteum* strain CMW 10309.

## Discussion

Resuscitation of the fungal endophytes from stock cultures was successful as morphology and pigmentation matched the original plates. In this study, extracts of darkly pigmented fungal endophytes had no activity against *P.* *aeruginosa*. A study by Sharma et al*.*^20^ described a fungus (*Pestalotiopsis* *neglecta*) with olive green pigmentation, which exhibited antibacterial activity for the tested strains with MIC values ranging from 6.25 to 25.00 µg/mL. The darkly pigmented fungus was found to contain several metabolites, which were supposedly responsible for antibacterial activity. However, in this study, ZF 52, ZF 91 and ZF 34, which were lightly pigmented had good activity against *P.* *aeruginosa*. This suggests that bioactivity of fungal endophytes depends largely on the nature of the compounds they biosynthesize. It is possible that some coloured metabolites of *P.* *neglecta* contributed largely to the antibacterial activity whilst colourless and/or less coloured active metabolites of ZF 52, ZF 91 and ZF 34 were responsible for their anti-*P.* *aeruginosa* activity.

The peak area relative integration of the annotated metabolites showed that compound **1** accounted for 12.68% of the crude fungal extract (2 g). The structure of compound **1** was elucidated based on the coherence in the various spectroscopic data obtained from the characterization procedures. The ^1^H NMR and ^13^C NMR chemical shifts showed the characteristic resonances of compound **1**, a terpene dilactone with molecular formula of C_16_H_18_O_4_ and an exact mass *m/z* 275.1283 [M + 1]^+^. The physical data of compound **1** afforded the structure of the C_16_-terpene dilactone, 3a,10b-dimethyl-1,2,3,3a,5a,7,10b,10c-octahydro-5,8-dioxa-acephenanthrylene-4,9-dione, which is consistent with that of CJ-14445 that was previously found in the fungus *Oidiodendron* *griseum*^14^ and synthetically obtained^21,22^. This compound has been previously found in *Botryosphaeria* sp., a fungal endophyte closely related to *N.* *luteum* isolated in the present study.

Compound **1** and its close relatives, yukonin and the oidiolactones were listed in the 2013 edition of Dictionary of Antibiotics and Related Substances^16^ following its earlier discovery from *O.* *griseum* and subsequent synthesis^22^ and antimicrobial studies^23^. The dilactone showed low activity against the tested bacterial strains (MICs were greater than 25 µg/mL) but better activity on fungal strains (MICs between 3.12 and 12.5 µg/mL). In other studies, the compound exhibited potential as antifungal but not as antibacterial agents^24,25^. In contrast, compound **1**, in the present study displayed good activity (0.61 µg/mL) against *P.* *aeruginosa*. This suggests that the fungi-derived dilactone may be selective to *P.* *aeruginosa*. At this stage, the mechanism of action of compound **1** against the tested bacterial strain is still unclear. Knowledge on the molecular dynamics of the compound on the respective protein architecture of the bacterial receptor will provide insight into the possible selectivity of the compound which is beyond the scope of the present study.

The extract of ZF 52, upon UPLC-ESI–MS analysis showed the presence of CJ-14445, a dilactone which has never been observed in *K.* *africana*. Being a C-_16_ compound, the biosynthesis must have proceeded via cyclization of farnesyl pyrophosphate (FPP) whose synthases is probably available significantly in the fungus in comparison with the host plant. However, FPP is involved in the build-up of other precursors for diterpenes and triterpenes found in *K.* *africana*, with associated synthases occurring in the fungus and host plant. This is likely responsible for the presence of limonoids like kigelianolide and 1-*O*-deacetyl-2α-hydroxykhayanolide E (biosynthetically derived from the triterpene skeleton) in ZF 52 and previously from *K.* *africana*^26^. Other compounds identified in ZF 52 are 6-*p*-coumaroylsucrose, martinoside and oxidized verbascoside. These are coumaric acid derivatives, a compound class that has been found in *K.* *africana*^27^. The similarity in previously isolated phytocompounds from *K.* *africana* and those identified in the fungus ZF 52 by UPLC-ESI–MS supports the inference that the endophyte has potential to biosynthesize metabolites produced by its host plant, as earlier observed for compounds like paclitaxel, camptothecin, podophyllotoxin, among others^1^.

Sequencing and BLAST analysis of the ITS2 amplicon of ZF 52 showed 99% identity to *N.* *luteum* strain CMW 10309. The last decade features literature on the isolation of endophytic fungi from *K.* *africana* but without mention of *N.* *luteum*. Idris et al.^2^ identified *Aspergillus* *flavus*, *Aspergillus* sp., *Cladosporium* sp. and *Curvularia* *lunata* as well as three unknown species from the leaves, fruits and bark of the plant. *Colletotrichum* sp was further obtained from the leaves, in another study^17^. The stem bark of *K.* *africana* yielded *Penicillium* *nigricans*^28^ and *Botryosphaeria* *dothidea*^29^. The largest (25 species) fungal population was reported from the leaves of *K.* *africana* collected during summer and winter^30^, providing a comprehensive catalogue of the mycobiome in the plant. Although *N.* *luteum* has not previously been isolated from *K.* *africana*, it is known to inhabit other plant species of different genera and families, most of which have a parasitic relationship, with varying degree of pathogenicity^31–33^. Bodede et al.^34^ reported *Neofusicoccum* sp*.* GT4 (KC507279.1) from the leaves of *Zanthoxylum* *capense* collected at a similar geographical location to the present study. Distribution of endophytic fungi in plants is thus dependent on several environmental factors including the genetic make-up and microenvironment of the plant.

*Neofusicoccum* *luteum* and species of the Botryosphaeriaceae family have been reported to produce different metabolites, most of which are low molecular weight phenolics, linked with fungal phytotoxicity^11^. The phytotoxic metabolites of *N.* *parvum* were reported to belong to the chemical families dihydroisocoumarin, dihydrotoluquinone, epoxylactone and hydroxybenzoic acid^35^. In the present study, chemical scaffolds containing lactone, phenylpropanoid, phenylethanoid, bonded sugar moieties and the limonoids’ furanylsteroidal skeleton were represented in the identified metabolites of *N.* *luteum*.

In conclusion, isolation of the fungal endophyte, *N.* *luteum*, was reported for the first time from the medicinal plant *K.* *africana*. The C_16_-terpene dilactone (CJ-14445) was identified as the major bioactive metabolite from the ethyl acetate extract of the fungus. Our finding shows that CJ-14445 is a potential antimicrobial candidate for *P.* *aeruginosa*, a resistant and lethal microbe. This study provides an alternative and sustainable biotechnological approach to harvest the antimicrobial dilactone, CJ-14445. Further studies will be required to understand the mechanism of action of the dilactone’s antibacterial activity against *P.* *aeruginosa*.

## Materials and methods

### General experimental procedures

All organic solvents (analytical grade) and other chemicals used in this study were supplied by either Merck (Darmstadt, Germany) or Sigma (St. Louis, USA) chemical companies. Merck 20 cm × 20 cm silica gel 60 F_254_ aluminium sheets were used for thin layer chromatography (TLC). Developed TLC plates were viewed under an ultraviolet lamp (254 and 366 nm) and further visualized with 10% H_2_SO_4_ in MeOH followed by heating^36^. Column chromatography (CC) was carried out using Merck silica gel 60 (0.040–0.063 mm). ^1^H, ^13^C and 2D nuclear magnetic resonance (NMR) spectra were recorded using deuterated chloroform (CDCl_3_) at room temperature on a Bruker Avance^III^ 400 MHz spectrometer.

### Fungal culture for biomolecule production

The leaves of the plant species (*K.* *africana*) were collected from the grounds of the Westville campus, University of KwaZulu-Natal (UKZN), South Africa. Permission to sample the leaves for research purposes was not required for registered students and staff of the institution. However, the experiments carried out on the *K.* *africana* in this study do not only complied with guidelines of UKZN but also South Africa and international guidelines on plant-based research. The plants were identified and authenticated by Dr Syd Ramdhani who deposited the voucher specimen in the ward herbarium at the School of Life Sciences, UKZN. Plant leaves collected were pre-treated according to standard protocols. Briefly, the leaves were plucked, packed in a well perforated bag and air dried at room temperature for 2 weeks. The dried leaves were crushed and kept in an airtight polyethylene bag until further analysis. The leaves were subjected to surface sterilization using the method of Kaewkla and Franco^37^. Potato dextrose agar (PDA) and rose bengal agar (RBA) were autoclaved for 15 min at 121 °C and then cooled to 40–50 °C for 20 min. The agar media were prepared with or without antibiotic supplements (chloramphenicol, ampicillin or streptomycin) and then poured into 9 cm Petri dishes. Thereafter, plates were inoculated with the sterilized *K.* *africana* leaf segments (length 1 cm^2^, 3 explants per plate), in triplicate, incubated at 25 °C. Pure fungal isolates were cultured and identified based on morphological characterization.

Forty-eight pure fungal isolates were sub-cultured onto potato dextrose agar (PDA). The isolates were grown for four days at room temperature (RT), stored at 4 °C and used as working stocks^38^. Long-term endofungal stocks were prepared by inoculating spores from aged plates into 1.5 mL Eppendorf tubes containing 1 mL of 40% glycerol. To induce production of biomolecules, standardized inocula (8 mm blue pipette tip back) from PDA plates were inoculated into rice medium (10 g of rice supplemented with 20 mL peptone water (0.5% peptone and 0.3% sodium chloride)). Solid substrate fermentation (SSF) was performed at room temperature for 30 days^39^. In addition, to determine optimum time of bioactive molecule production, aliquots of biomass were extracted weekly, for four weeks.

### Solvent extraction of bioactive molecules

Biomolecules were extracted from mycelial mats using EtOAc^40^. To halt the fermentation process, 10 g of the rice medium containing the biomass was soaked in 50 mL of EtOAc for 24 h under gentle agitation (100 rpm, RT). The biomass and the EtOAc extract were separated by vacuum filtration through Whatman No.1 filter paper. The EtOAc extract containing bioactive compounds was washed with 50 mL demineralized water to remove traces of starch. The organic layers were collected and evaporated using a rotary evaporator. The extract was weighed and resuspended in EtOAc to a final concentration of 10 mg/mL and stored in the refrigerator at 4 °C until required.

### Evaluation of anti-P. aeruginosa activity

The disc diffusion assay was carried out using the method described by Alderman and Smith^41^. The test bacterium, *P.* *aeruginosa* ATCC 27853 was inoculated onto nutrient agar (NA) plates and incubated for 24 h at 37 °C. For a standardized inoculum for the disc diffusion assay, culture was inoculated in 500 µL of demineralized water and the cell density was standardized to 0.5 McFarland standard using a spectrophotometer (UV 1800). The standardized bacterial cells were spread onto Mueller–Hinton (Merck, Germany) plates using sterile cotton swabs and left to dry. Extracts (100 µL) were saturated on sterile filter paper discs and used as antibiotic discs. The plates were incubated at 37 °C for 24 h and the resultant zones of inhibition were measured and scored according to Chenia (2013)^42^. Furthermore, minimum inhibitory concentrations (MICs) were determined by the method described by Jorgensen (1999)^43^ with slight modification.

### Compound isolation, purification and identification

A preliminary TLC profile of the crude extract was obtained by spotting it onto TLC plates and developed in various solvent systems of chloroform (CHCl_3_):methanol (MeOH) and hexane:EtOAc (using 100% CHCl_3_ or hexane that was increased stepwise by 10% to 100% MeOH or EtOAc, respectively) to assess their separation efficiency^44^.

The EtOAc extract (2 g) of ZF 52 was subjected to CC using a glass column (length: 53 cm and width: 15 cm) and separation achieved using hexane:EtOAc as the mobile phase, starting with 100% hexane. The polarity of the solvent system was increased by 5% after 100 mL of eluate was collected for each eluent step, until 100% EtOAc was reached. The polarity of the mobile phase was further increased with 5 and 10% MeOH, sequentially. A total of 27 aliquots were collected and combined into four fractions, namely, A (1–10, 230 mg), B (11–16, 310 mg), C (17–21, 150 mg) and D (22–27, 310 mg), based on similarities in the TLC profiles of the aliquots. Fraction C afforded compound **1** upon crystallization from ethanol and its structure was determined using 1 and 2D NMR spectroscopy and single crystal XRD analysis.

### Analysis of ZF 52 using Ultra Performance Liquid Chromatography-Electrospray Ionization-Mass Spectrometry (UPLC-ESI–MS)

The EtOAc extract of ZF 52 was analysed using a Waters UPLC coupled in sequence to Waters SYNAPT™ HDMS™ system (Waters Corporation, MA, USA). An optimized chromatographic separation was obtained on Waters UPLC utilizing a Waters HSS T3 C18 column (150 mm × 2.1 mm, 1.8 µm), temperature set at 60 °C and a binary solvent mixture of water (Eluent A) and acetonitrile (Eluent B) both containing 10 mM formic acid (pH of water adjusted to 2.4) was used. Initial condition was 98% A at a flow rate of 0.4 mL/min, maintained for 1 min, followed by a linear gradient to 2% A at 16 min. Firstly, the condition was kept constant for 1 min and then changed to the initial condition. The runtime was 20 min while the injection volume was 2 µL. The sample was then maintained at 8 °C.

A SYNAPT G1 mass spectrometer, used in V-optics and operated in electrospray mode allowed detection of all compounds compactible with electrospray ionization (ESI). Leucine enkephalin (50 pg/mL) was used as a reference calibrant to obtain mass accuracies between 1 and 5 mDa. The ESI positive mode was obtained from the mass spectrometer (capillary voltage of 2.5 kV, sampling cone at 30 V, extraction cone at 4.5 V). The scan time was 0.1 s covering the 50–1000 Dalton mass range with an interscan time of 0.02 s. Source temperature was 120 °C and desolvation temperature was set at 450 °C. Nitrogen gas was used as the nebulization gas at a flow rate of 550 L/h and cone gas was added at 50 L/h. The massLynx 4.1 (SCN 872) software was employed to control the hyphenated system and for data processing. Compounds identification was further enhanced by analyzing all samples with both low and high collision energy settings of the collision cell. Low energy setting of 3 eV was used (to reduce fragmentation of compounds), while the collision energy ramp was in the range of 10–40 eV for molecule fragmentation. Argon was used as the collision gas in the collision cell. All possible compounds were ionized, and their respective peaks integrated and annotated.

### Molecular identification of fungal isolate producing compounds of interest

Genomic deoxyribonucleic acid (DNA) isolation was conducted on ZF 52 using the ZR Soil Microbe DNA Miniprep™ (Zymo Research, USA) according to the manufacturer’s instructions. After extraction, the DNA was analysed by agarose gel electrophoresis. DNA samples were stored at − 20 °C. The universal primers for the fungal internal transcribed spacer two (ITS2) region of the 18S ribonucleic acid (rRNA) gene were used in the polymerase chain (PCR) reaction^45,46^ while the ITS primer pair sequences were ITS 5 (5' - GGAAGTAAAAGTCGTAACAAGG - 3') for the forward and ITS 4 (5' – TCCTCCGCTTATTGATATGC - 3') for the reverse. The PCR reaction mixture (50 µL) consisted of template DNA, 2.5 µM of each of the forward and reverse primers, 25 mM MgCl_2_, 10 mM dNTPs, Taq DNA polymerase, 10 mM buffer (Thermo Scientific). The reaction mixture was brought to 50 µL using double distilled water. Amplification of the reaction mixture was conducted under the following thermal cycling conditions: initial denaturation at 94 °C for 5 min, 30 cycles of denaturation at 94 °C for 30 s, annealing at 55 °C for 30 s and extension at 72 °C for 30 s^41^. The PCR products were visualized using electrophoretic analysis on a 1% agarose gel stained with ethidium bromide. After confirmation that the amplification was successful, amplicons were sequenced using Sanger sequencing at Stellenbosch University, South Africa. The identity of the isolates was determined using the basic local alignment search tool (BLAST) analysis, on the NCBI database^47^.

## Supplementary Information

Supplementary Information.
