# Supplementary material for: Anti-Pseudomonasaeruginosa activity of a C16-terpene dilactone isolated from the endophytic fungus Neofusicoccumluteum of Kigeliaafricana (Lam.)
Source: Sci Rep. 2022 Jan 17;12:780. doi: 10.1038/s41598-021-04747-x (PMC8763916; doi:10.1038/s41598-021-04747-x)
Supplement: Supplementary file 1 — Supplementary Information. [file 41598_2021_4747_MOESM1_ESM.pdf]

Anti-*Pseudomonas aeruginosa* activity of a C_{16}-terpene dilactone isolated from the endophytic fungus *Neofusicoccum luteum* of *Kigelia africana* (Lam.)

Olusola Bodede[1,3], Mamokoena Kuali[2], Gerhard Prinsloo[3], Roshila Moodley[1], Roshini Govinden[2]

[1]School of Chemistry & Physics, University of KwaZulu-Natal, Westville Campus. Private Bag X54001, Durban, 4000, South Africa.

[2]School of Life Sciences, University of KwaZulu-Natal, Westville Campus. Private Bag X54001, Durban, 4000, South Africa.

[3]Department of Agriculture and Animal Health, University of South Africa, Florida Campus, Florida, 1710, South Africa.

* Correspondence: 🖂 ebodedos@unisa.ac.za

Supplementary material

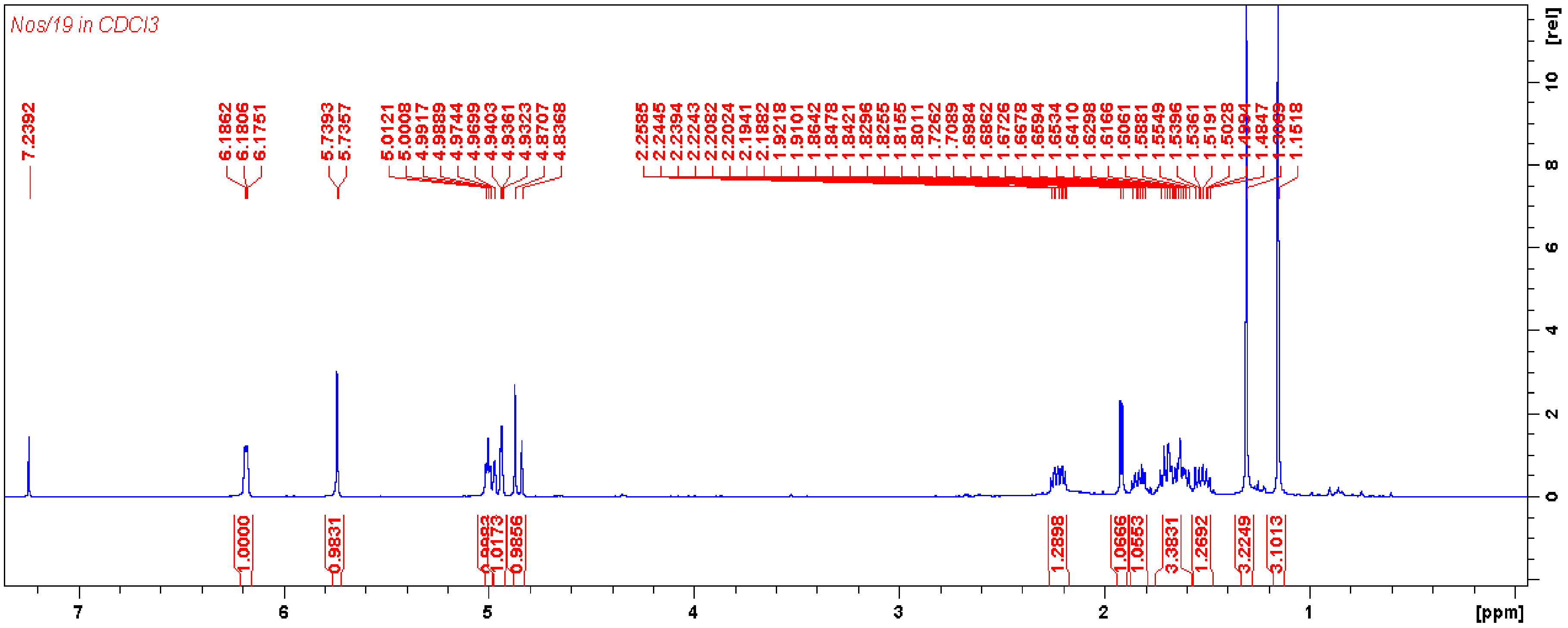

Figure S1. ^{1}H NMR spectrum of compound **1.**

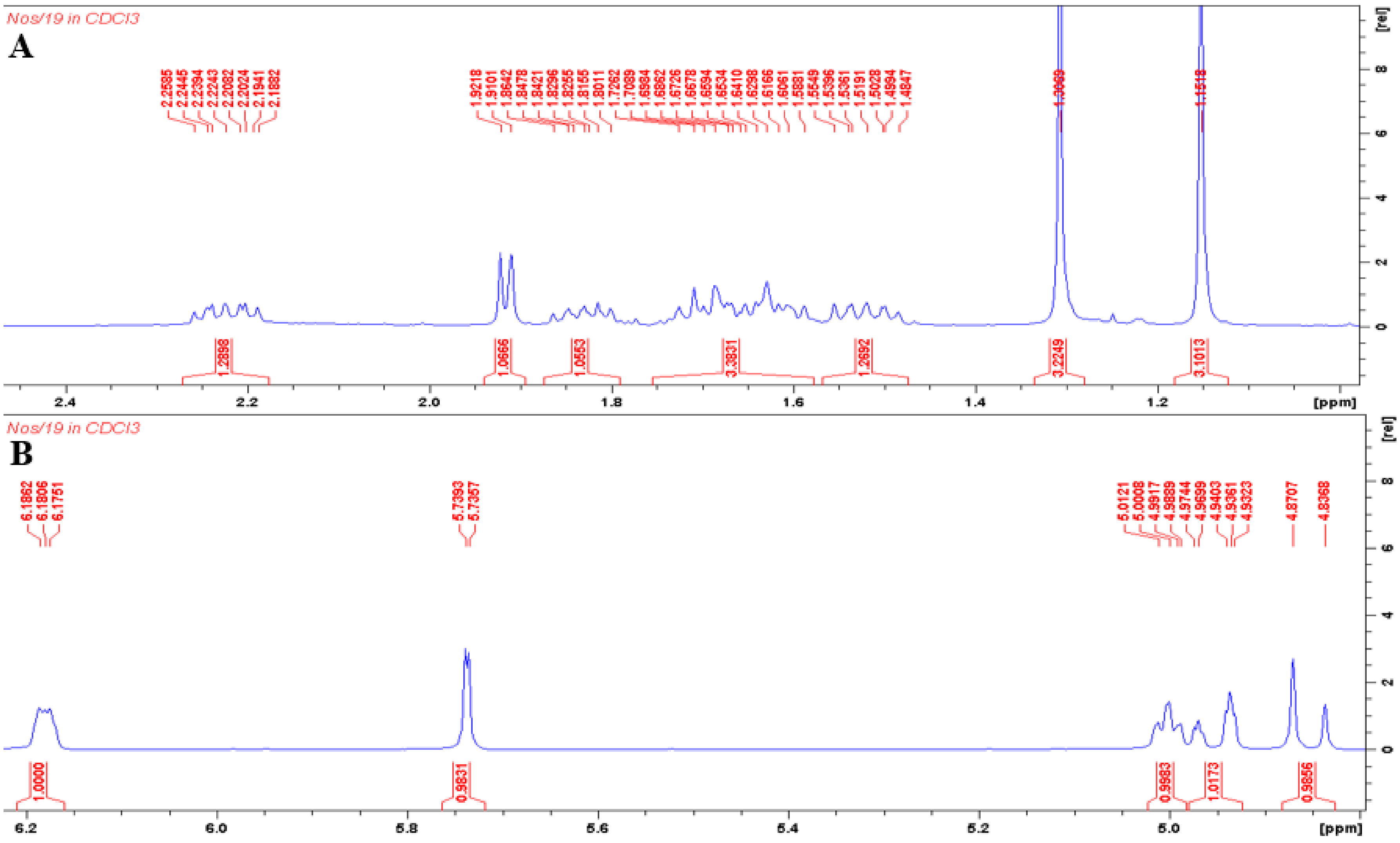

Figure S2. Expanded ^{1}H NMR spectrum of compound **1.**

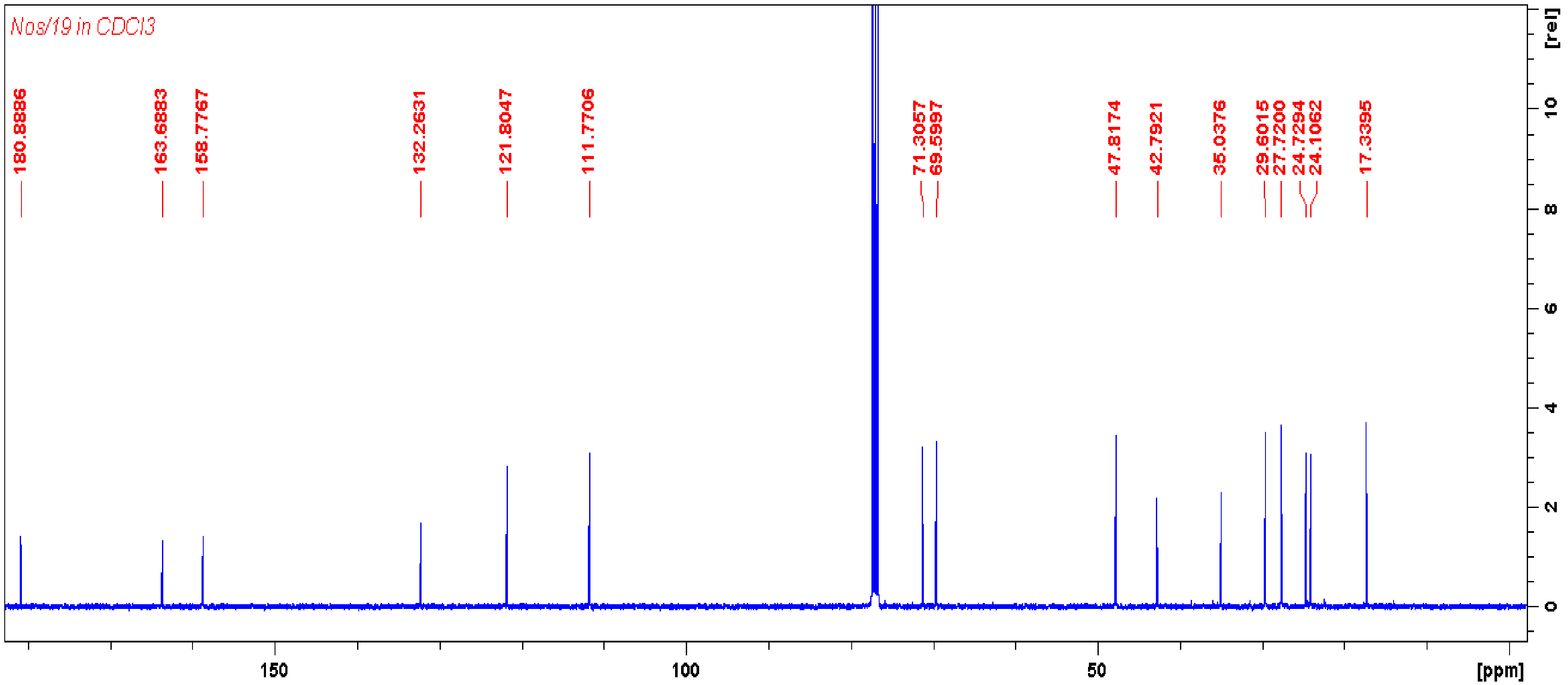

Figure S3. ^{13}C NMR spectrum of compound **1.**

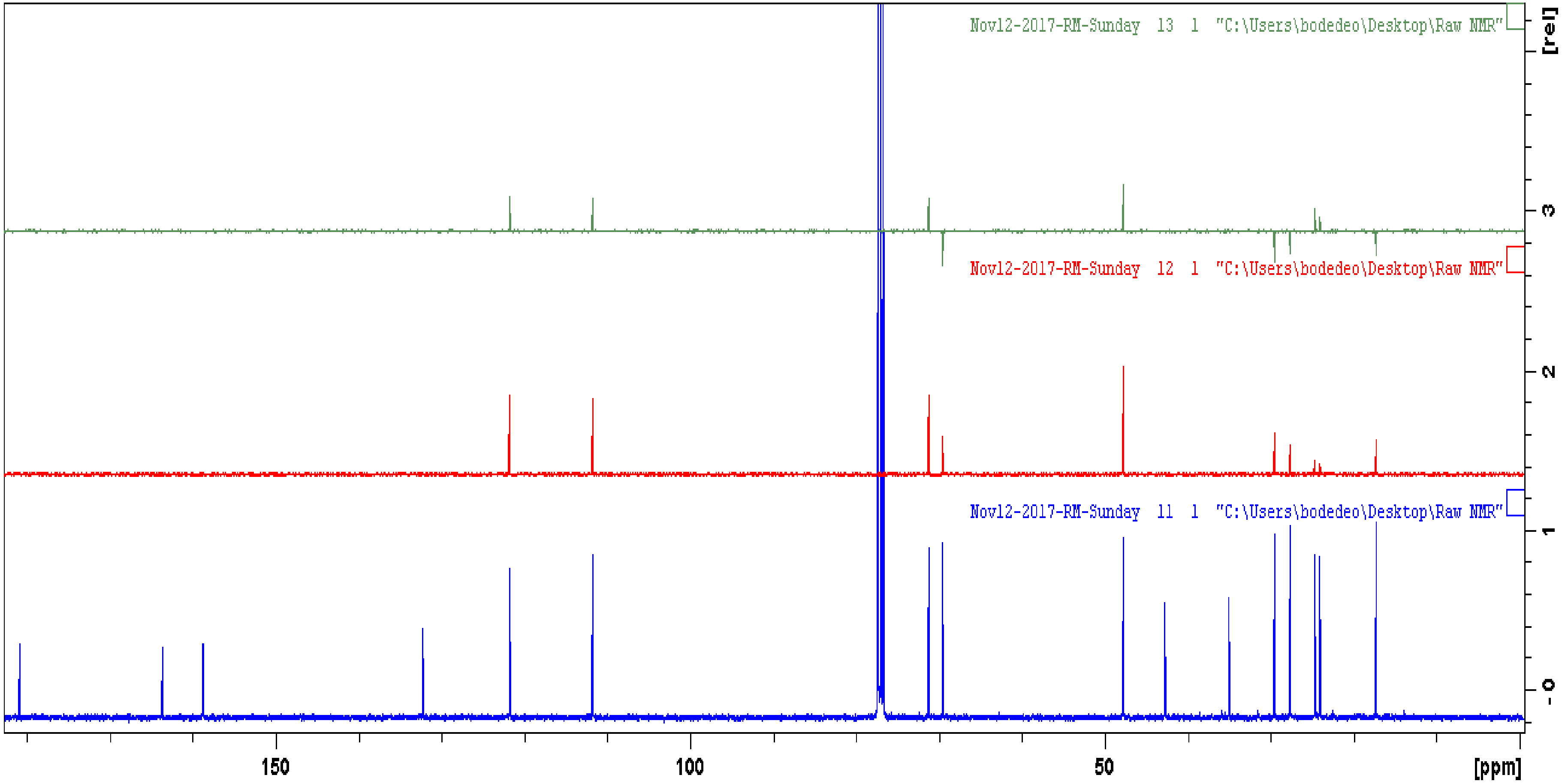

Figure S4. DEPT spectrum of compound **1.**

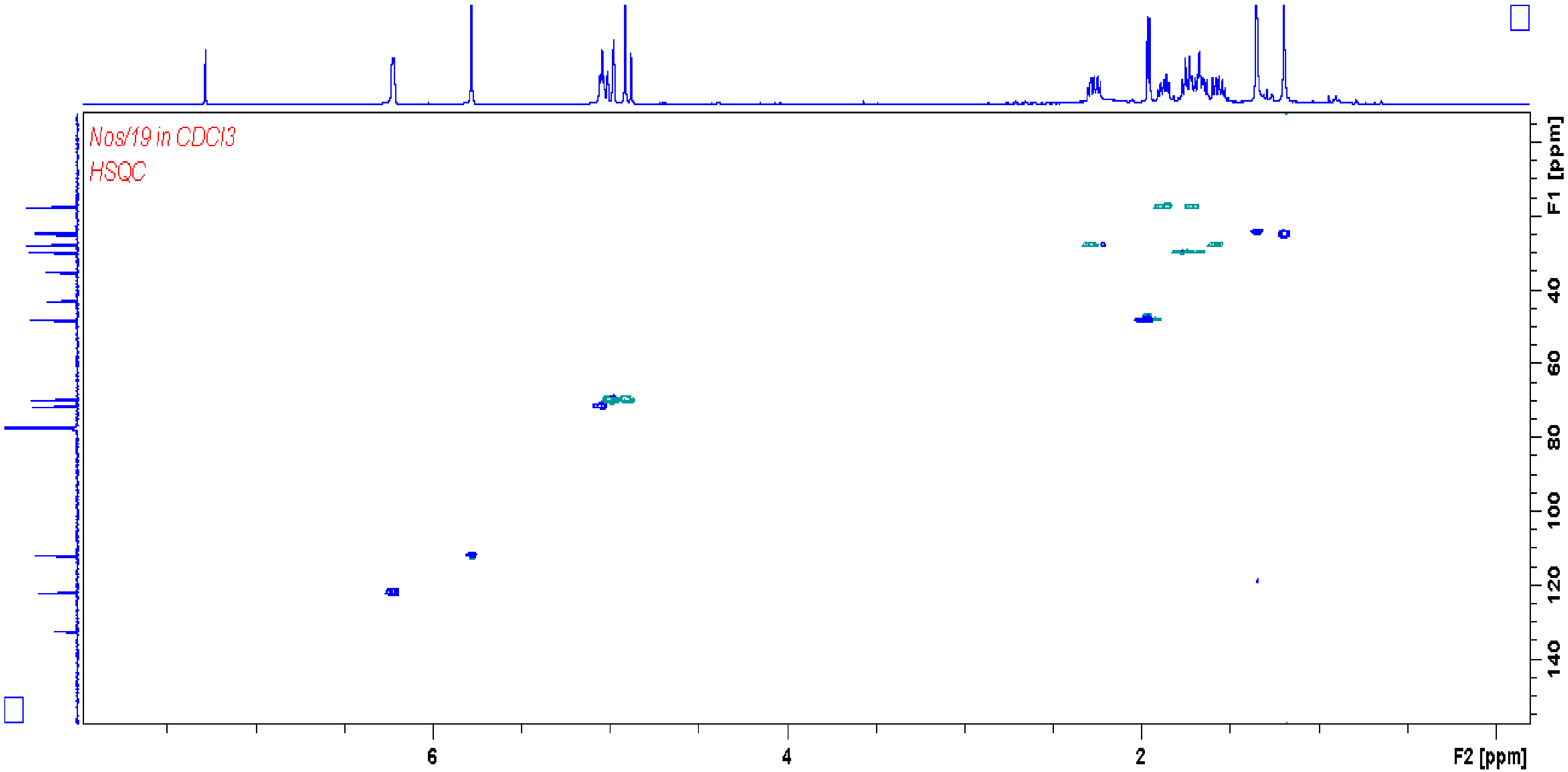

Figure S5. HSQC spectrum of compound **1.**

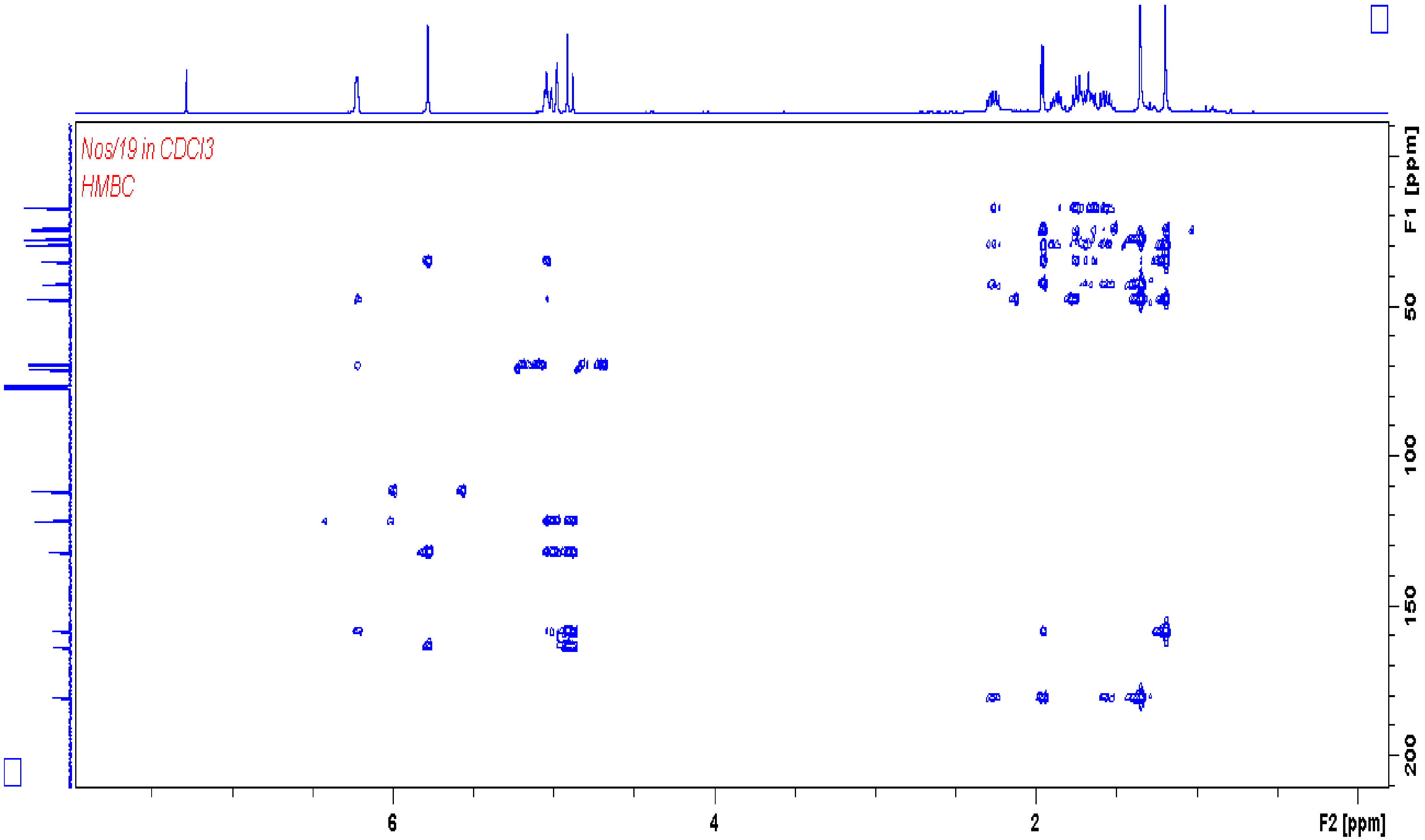

Figure S6. HMBC spectrum of compound **1.**

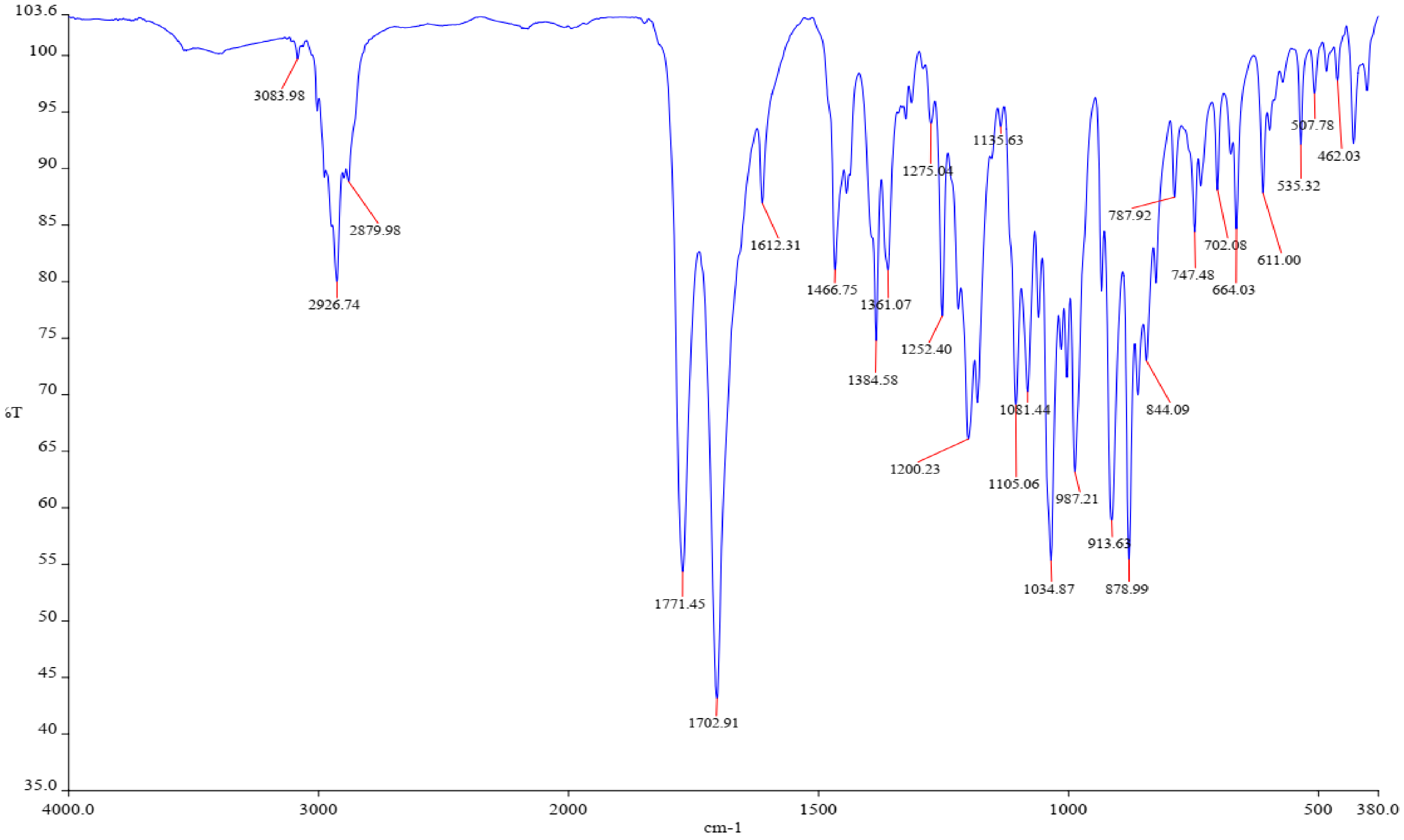

Figure S7. Infrared spectrum of compound **1.**

Elemental Composition Report

Single Mass Analysis
Tolerance = 5.0 mDa / DBE: min = -1.5, max = 100.0
Element prediction: Off
Number of isotope peaks used for i-FIT = 3

Monoisotopic Mass, Even Electron Ions
40 formula(e) evaluated with 1 results within limits (up to 10 best isotopic matches for each mass)
Elements Used:
C: 1-50 H: 1-100 O: 1-20

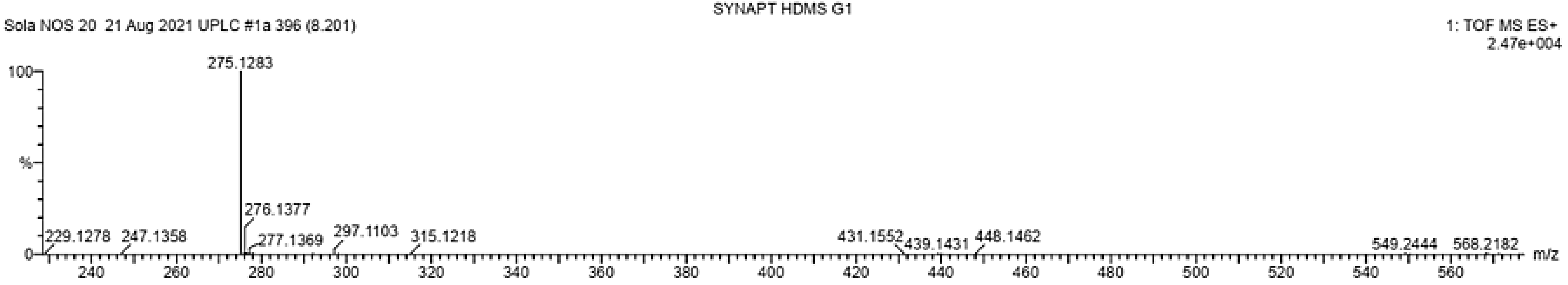

Minimum:				-1.5			
Maximum:		5.0	10.0	100.0			
Mass	Calc. Mass	mDa	PPM	DBE	i-FIT	i-FIT (Norm)	Formula
275.1283	275.1283	0.0	0.0	7.5	118.1	0.0	C16 H19 O4

Figure S8. HRMS spectrum of compound **1.**

Single crystal XRD report

Datablock: 18zs_bm_s1_0ma

```
Bond precision:  C-C = 0.0031 A         Wavelength=0.71073

Cell:            a=18.120(3)     b=7.6922(13)        c=12.393(4)
                 alpha=90        beta=129.372(6)     gamma=90
Temperature:     100 K

                Calculated                Reported
Volume          1335.3(5)                 1335.3(5)
Space group     C 2                       C 1 2 1
Hall group      C 2y                      C 2y
Moiety formula  C16 H18 O4                C16 H18 O4
Sum formula     C16 H18 O4                C16 H18 O4
Mr              274.30                    274.30
Dx,g cm-3       1.365                     1.364
Z               4                         4
Mu (mm-1)       0.097                     0.097
F000            584.0                     584.0
F000'           584.32
h,k,lmax        24,10,16                  24,10,16
Nref            3347[ 1794]               3281
Tmin,Tmax       0.979,0.989               0.464,0.746
Tmin'           0.972

Correction method= # Reported T Limits: Tmin=0.464 Tmax=0.746
AbsCorr = MULTI-SCAN

Data completeness= 1.83/0.98      Theta(max)= 28.338

R(reflections)= 0.0387( 3112)     wR2(reflections)= 0.1032( 3281)

S = 1.061                 Npar= 183
```

Alert level C

PLAT911_ALERT_3_C	Missing FCF Refl Between Thmin & STh/L=	0.600	3 Report

Alert level G

PLAT032_ALERT_4_G	Std. Uncertainty on Flack Parameter Value High .	0.300 Report
PLAT128_ALERT_4_G	Alternate Setting for Input Space Group C2	I2 Note
PLAT398_ALERT_2_G	Deviating C-O-C Angle From 120 for O3	109.1 Degree
PLAT791_ALERT_4_G	Model has Chirality at C5 (Chiral SPGR)	S Verify
PLAT791_ALERT_4_G	Model has Chirality at C9 (Chiral SPGR)	S Verify
PLAT791_ALERT_4_G	Model has Chirality at C10 (Chiral SPGR)	R Verify
PLAT791_ALERT_4_G	Model has Chirality at C12 (Chiral SPGR)	R Verify
PLAT912_ALERT_4_G	Missing # of FCF Reflections Above STh/L= 0.600	15 Note
PLAT978_ALERT_2_G	Number C-C Bonds with Positive Residual Density.	13 Info

0 **ALERT level A** = Most likely a serious problem - resolve or explain
0 **ALERT level B** = A potentially serious problem, consider carefully
1 **ALERT level C** = Check. Ensure it is not caused by an omission or oversight
9 **ALERT level G** = General information/check it is not something unexpected

0 ALERT type 1 CIF construction/syntax error, inconsistent or missing data
2 ALERT type 2 Indicator that the structure model may be wrong or deficient
1 ALERT type 3 Indicator that the structure quality may be low
7 ALERT type 4 Improvement, methodology, query or suggestion
0 ALERT type 5 Informative message, check

PLATON version of 30/01/2018; check.def file version of 30/01/2018

Datablock 18zs_bm_s1_0ma - ellipsoid plot

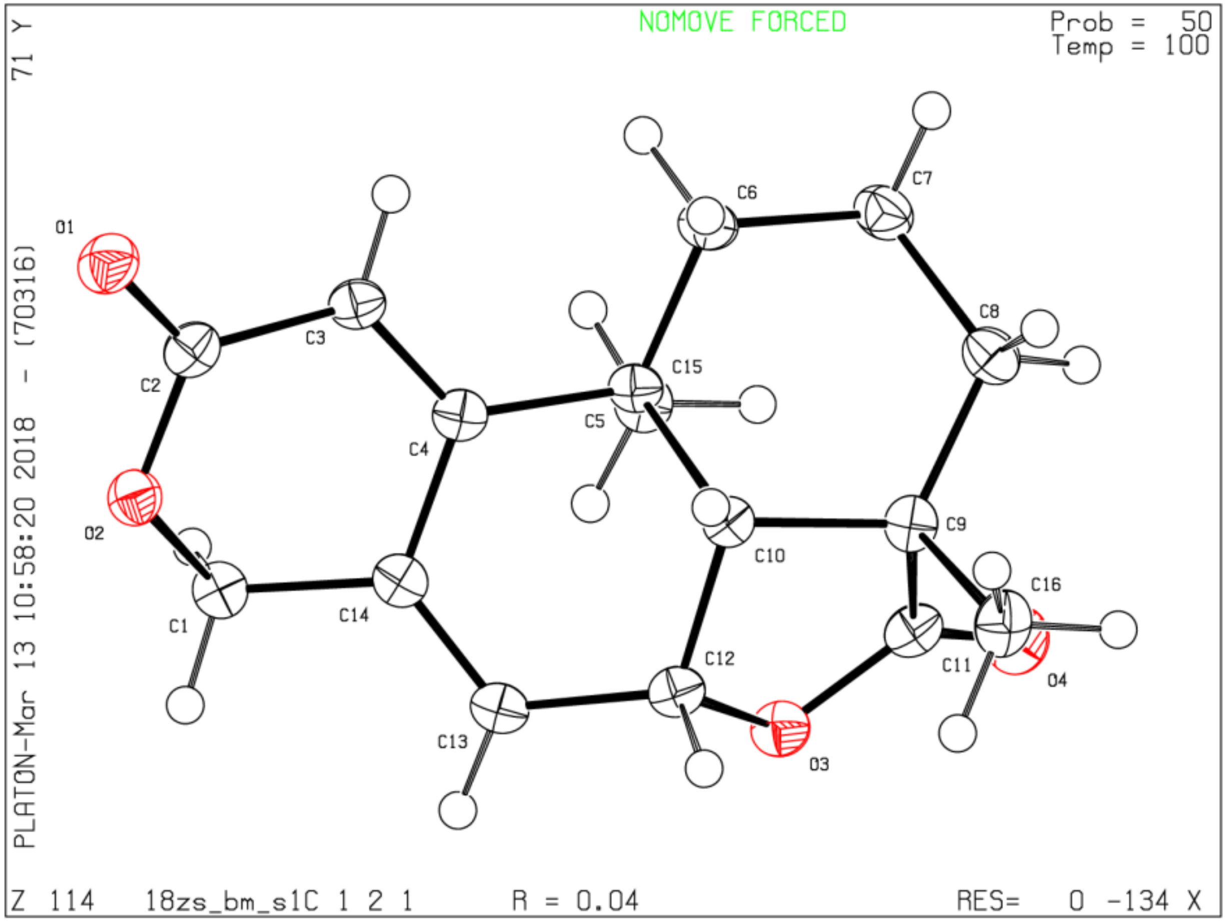

Figure S9. Crystal structure of compound **1.**

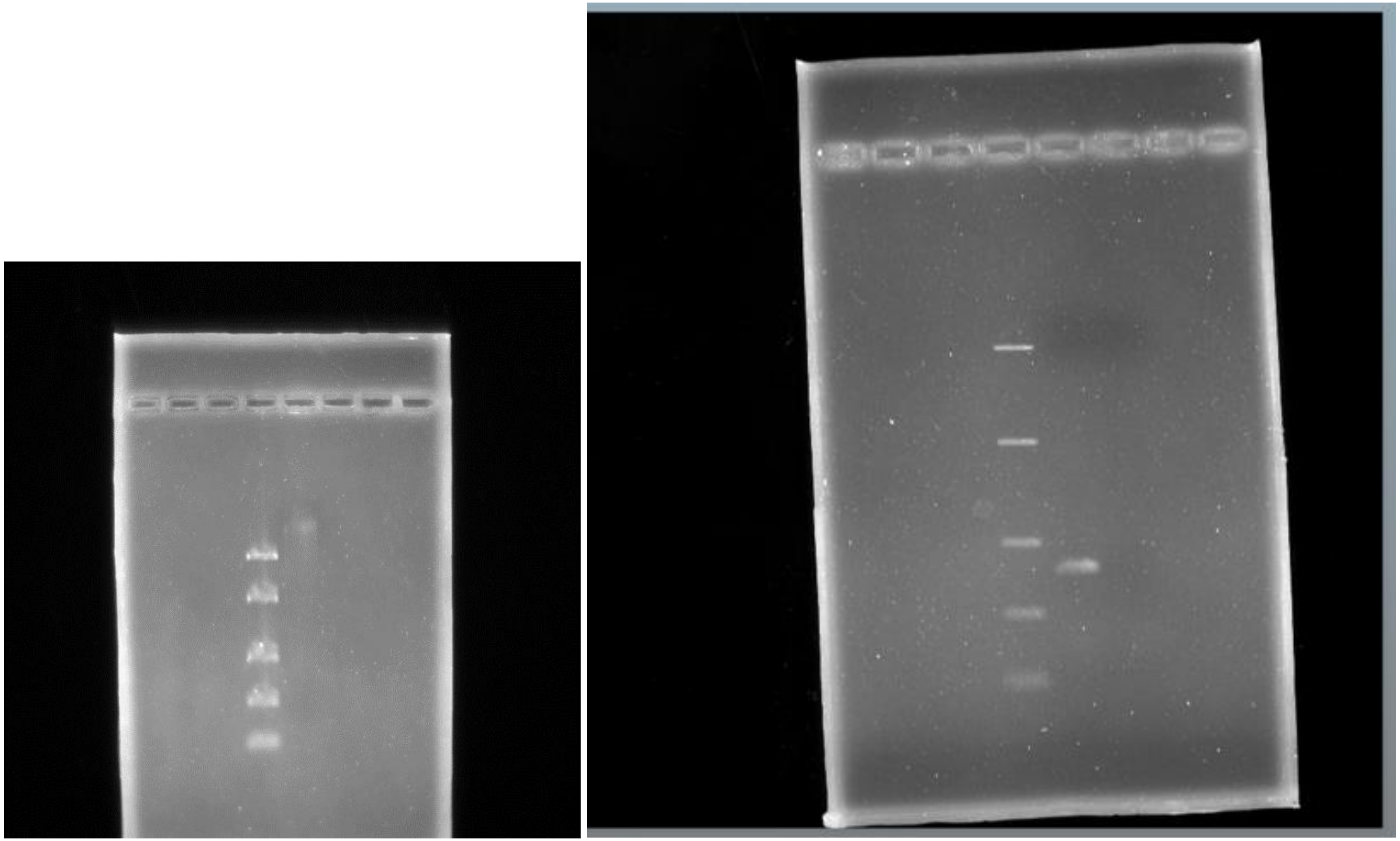

Figure S10. Original, unprocessed images of gels (genomic DNA – left, amplification product – right).
